# On Eupatorium and Serpentaria

**Published:** 1864-09

**Authors:** M. B. Beck

**Affiliations:** P. A. C. S.


ART. IX.—*On Eupatorium and Serpentaria.* By Dr. M. B. BECK, P. A. C. S.

In bringing these two valuable remedies before the eyes of the medical profession, I have scarcely anything *new* or *exciting* to say about them; for I am aware that many of our best physicians have been for many years, and are now, using them, in various diseases, with beneficial results; also their properties and their *modus operandi* have been, from time to time, ably and fully set forth by experienced and competent men. My chief object is to ask not only for these, but for all our valuable *indigenous medicines*, a more extended and general use, especially when other remedies are difficult to be obtained, and command so high a price.

The boneset, eupatorium and the snake root, (serpentaria Virginiana,) have long been held in great repute, particularly in the treatment of all fevers of a low grade, whether intermittent, remittent or continued; and it is especially for these diseases, or the so-called typhoid and camp fevers, that I would press their merits upon the attention of the profession.

About two years ago, I had some eight or ten cases *at one time*, on a farm, of what is generally called typhoid fever, and, after giving some mild mercurial, I used a cold decoction—I prefer the decoction to the infusion—in the proportion of about half an ounce of the dried leaves of the boneset and the same quantity of snake root to a pint of water, giving of that a wineglassful every three or four hours, day and night, pro. re nata, with entire success; that is, I mean to say I used no other tonic, and all the patients had a good recovery. This decoction, as far as my limited experience goes, can be given when neither quinine nor cinchona may be admissible; it also makes an admirable menstrum for the tr. cinch. comp.; and just here allow me to say that, in my opinion, the tr. cinch. comp. would be greatly improved by a little more of the serpentaria in it. Let me then respectfully ask of all those who have more extended means and better opportunities, to give these and all our indigenous remedies a fair and full trial, and report their various experiences.

The eupatorium is now in the proper condition to be gathered, and should be dried on the stalk in an attic, *dry* and *dark*; for snake root, the month of September is best. It is very probable to my mind, that the want of success and confidence in many of our remedies is owing, in a great degree, to the improper time at which they are collected, and to the wrong way of curing or preserving them when collected, and, lastly, to the inefficacious manner in which they are prepared or compounded for administration. These are not matters of small importance, and I hope that the framers of our "Confederate States Dispensatory," should we have one, will give all these points a more careful attention than they have heretofore received.

